# Surgically cured hypoglycemia secondary to pleural solitary fibrous tumour: case report and update review on the Doege-Potter syndrome

**DOI:** 10.1186/1749-8090-4-45

**Published:** 2009-08-18

**Authors:** Ahmed Y Kalebi, Martin J Hale, Michelle L Wong, Tessa Hoffman, Jill Murray

**Affiliations:** 1 Department of Anatomical Pathology, National Health Laboratory Service [NHLS], Johannesburg, South Africa; 2 Division of Pulmonology, University of the Witwatersrand, Johannesburg, South Africa; 3 School of Public Health, National Institute of Occupational Healths, Johannesburg, South Africa

## Abstract

The association of paraneoplastic hypoglycemia [Doege-Potter syndrome] and finger clubbing [Pierre-Marie-Bamberg syndrome] with pleural solitary fibrous tumour is rare. We present a previously unpublished but typical example of this rare occurrence together with a detailed updated literature review of previously published cases of pleural SFT discussing the histopathology of SFT; pathophysiology of the hypoglycemia and finger clubbing; treatment and outcome of pleural SFT. The patient, a 57-year-old African male was admitted at our hospital with recurrent episodes of hypoglycemia. He was found to have digital clubbing and decreased breath sounds in the right lower chest but no other significant clinical findings. His insulin level measured during an episode of hypoglycemia was undetectable. Chest radiograph and CT-scan revealed a lobulated mass in the right chest which was diagnosed to be SFT on histology. Surgical excision of the mass resulted in cure of the hypoglycemic episodes and rapid regression of the clubbing. Less than 65 cases of pleural SFT manifesting with hypoglycemia with or without finger-clubbing have been published in the English literature. The mean diameter of these tumours manifesting with hypoglycemia is 20 cm, 54% being benign while 42% were malignant. They predominantly present in the 6th-8th decade, average age of 64 years and a slight male preponderance at 58%. Complete surgical resection remains the most important predictor of clinical outcome in terms of recurrence and metastases, while providing instant cure for the hypoglycemia and rapid resolution of the finger clubbing.

## Background

The occurrence of hypoglycemia with an intrathoracic tumour was first reported by Doege and Potter independently in 1930 hence the eponym Doege-Potter syndrome [DPS] [1,2]. Hyperthrophic osteoarthropathy clinically observed as finger-clubbing is also associated with intrathoracic tumours and goes with the eponym of Pierre-Marie-Bamberg syndrome [PMBS]. In this report we present a previously unpublished but very typical example of the rare occurrence of hypoglycaemia and finger-clubbing with pleural solitary fibrous tumour [SFT] in an adult African male. A detailed updated literature review is presented detailing the histopathology of SFT, pathophysiology of the hypoglycemia and finger clubbing, treatment and outcome of pleural SFT.

## Case Presentation

**Figure 1:**
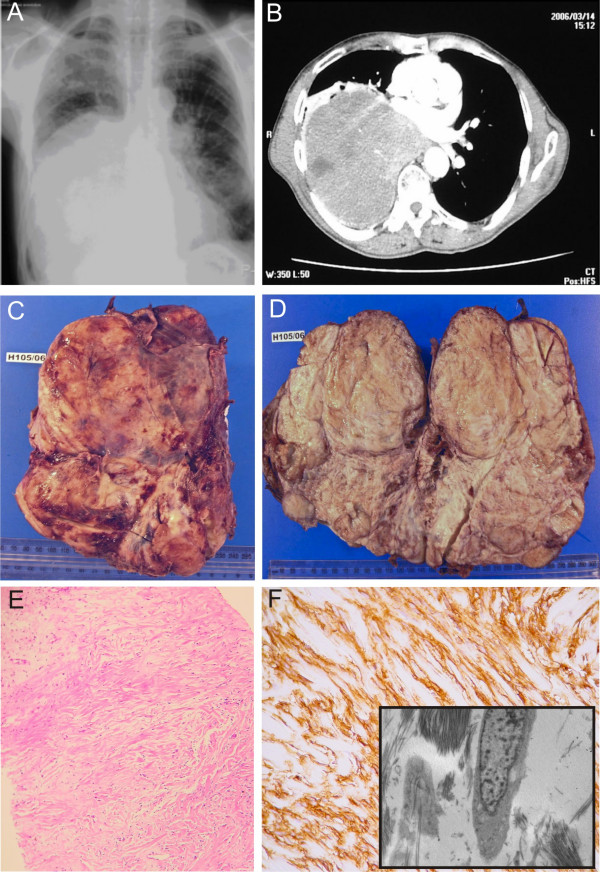
**1A: Chest radiograph showing a lobulated mass in the right chest suggestive of a benign pleural tumour**. 1B: CT-scan showing a lobulated mass in the right chest suggestive of a benign pleural tumour. 1C: Tumour at macroscopy, well-circumscribed and encapsulated. 1D: Cut surface of the tumour tan-coloured and whorled appearance. 1E: Photomicrograph showing bland spindle cell proliferation with a patternless architecture, and hypo- and hypercellular areas separated by dense collagenous fibrous stroma (H&E stain). 1F: Photomicrograph of tumour cells stained positive with CD34. 1F-inset: Electromicrograph depicting ultrastructural features of fibroblasts with abundant collagen.

A 57-year-old male was referred to the Chris Hani Baragwanath Hospital with recurrent episodes of symptoms of severe hypoglycemia including syncope. He had a 20 pack-year history of smoking. He was found on examination to have marked finger-clubbing and decreased breath sounds in the right lower chest, but no other significant clinical findings. Sputum cytology was negative for malignant cells. His blood glucose levels recorded over three consecutive days whilst remained low despite receiving continuous intravenous infusion of 10% dextrose (table 1). His serum insulin and C-peptide levels were also depressed (table 2). A chest radiograph and CT-scan showed a lobulated mass in the right chest suggestive of a benign pleural tumour (figure 1A-B).

**Table 1:** Patient's blood glucose recorded over three consecutive days from admission

**Time**	**Capillary blood glucose level (mmol/L)**
09 h 00	2.7

13 h 00	4.6

21 h 00	3.9

01 h 00	1.8

05 h 00	5.5

09 h 00	4.8

13 h 00	5.2

17 h 00	9.4

21 h 00	5.5

01 h 00	1.8

05 h 00	4.4

11 h 00	2.4

17 h 00	5.6

21 h 00	3.1

**Table 2:** Patient's serum insulin and C-peptide levels before surgery

Blood glucose:	2.2 mmol/L (4.1 - 11.1 mmol/L)
Insulin:	<2.0 mU/L (8.9-28.4)

C-peptide:	0.7 μg/L (1.1-5)

Cortisol:	394 nmol/L (250 - 850)

A percutaneous needle biopsy of the mass showed morphological and immunohistochemical features of a benign SFT. The patient underwent thoracotomy with excision of the bulk of the mass. A small remnant attached to the diaphragm could not be removed. The tumour was large, well-circumscribed and encapsulated, with a tan-coloured whorled cut surface (figure 1C-1D). It weighed 1744 g and measured 20 × 15 × 10 cm. There were no areas of intratumoral haemorrhage or necrosis.

Microscopic examination of the tumour revealed a bland spindle cell proliferation arranged in short intersecting fascicles with a patternless architecture, and hypo- and hypercellular areas separated by dense collagenous fibrous stroma (figure 1E). There was no evidence of cytological atypia or mitotic figures. Immunohistochemistry demonstrated that the tumour cells stained positive with CD34 (figure 1F) and Bcl-2, while negative for S100, CD68, CD99, Calretinin, h-Caldesmon, MSA and SMA. Electron microscopy demonstrated ultrastructural features of fibroblasts with abundant collagen (figure 1F-inset). A final diagnosis of pleural SFT was made.

The episodes of hypoglycemia abated postoperatively and the digital clubbing resolved within three months.

## Review

### Methodology of literature search

An extensive literature search on PUBMED was conducted using the key words SFT and its various synonyms to identify published cases of SFT. The search was then limited to reported cases of pleural based SFT with explicitly recorded hypoglycemia, with particular focus on the period from 1981 to 2008. Unfortunately some authors did not record whether or not extrathoracic symptoms were present in their series [3]. Data from cases prior to 1981 were primarily traced from the review by Briselli *et al *which had analysed 360 cases in the literature and 8 additional cases from their series [4]. All reports that were traced in the English literature were extracted and the full articles scoured through to ascertain the cases of pleuro-thoracic SFT with hypoglycemia with or without finger-clubbing. These cases were then tabulated and are herein discussed.

### Results from the literature search

We identified 48 cases of pleural SFT presenting with hypoglycemia that have been published since 1981 in the English literature. Briselli *et al *had previously reviewed 368 cases including their series of 8 cases from which they established that hypoglycemia was documented in 4% [4]. We therefore estimate that there are less than 65 cases of pleural SFT thus far published in the English literature.

Specific data available on the cases we reviewed since 1981 are outlined in table 3. Unfortunately individual data for patients with hypoglycemia was not recorded in some reports thus could not be analysed in this review [5-11] The patients' age range was 38 to 83 years, with an average of 64 years and a median of 65 years respectively. The majority of patients manifested hypoglycaemia in their 6^th ^to 8^th ^age decade. A slight male preponderance of 58% was noted. Interestingly we found that the youngest patient from our search, who happens to be the only under 40 years of age, was a 38 year old female who was diagnosed with 'pleural fibroma' while gravid at 13 weeks. Her hypoglycemia and finger clubbing resolved after surgery [12].

**Table 3:** Age, Gender, Weight, Size and Histology of pleural SFT in the literature [12-37]

Age (n = 30)	Mean	63.5 years
	
	Median	64.5 years
	
	Range	38-83 years
Gender (n = 40)	Males	23 (58%)
	
	Females	17 (43%)

Weight (n = 18)	Mean	2071 g
	
	Median	1822 g
	
	Range	850-4000 g

Maximum size (n = 24)	Mean	20.2 cm
	
	Median	20.0 cm
	
	Range	10-33 cm

Histological criteria (n = 39)	Benign	21 (54%)
	
	Malignant	18 (46%)

Finger clubbing (n = 40)	Recorded in 22 cases (55%)	

The average greatest dimension of these SFTs presenting with hypoglycemia as recorded since 1981 was 20 cm (table 3). England *et al *observed that 9/12 of the cases with hypoglycemia out of their series of 223 cases were greater than 10 cm in dimension [13]. The average weight of the tumours in our review was 2071 g with a range from 850 g to 4000 g (table 3). Malignant criteria [see under histopathology below] were fulfilled in 46% of the pleural SFT manifesting with hypoglycaemia while 54% were benign. Finger clubbing was noted in 22 cases [55%] whereby the observation was documented. All the cases that we reviewed from 1981 were surgically managed. Complete surgical removal was achieved in all but one case. Surgical removal led to long-term recurrence free survival together with resolution of hypoglycemia and finger-clubbing in essentially all the cases reviewed.

### Histopathology of SFT

Pleural SFT, first described as a distinct entity in 1931 [38], is an uncommon but not rare tumour. Well over 800 cases of pleural SFT have been reported in the medical literature, with many cases documented under synonyms or incorrect names [39-41]. Pleural SFT was considered to be of mesothelial origin, but recent evidence revealed it to be of mesenchymal histogenesis [42]. Extrathoracic SFT have thus been reported in the literature from sites such as the abdominal cavity, retroperitoneum, female genital tract, urinary tract and other sites, some of which may also be associated with hypoglycaemia [43-48].

At histology, pleural SFT is seen as a fibrous and myofibroblastic spindle cell neoplasm exhibiting a characteristic 'patternless' architecture, a typical hemangiopericytoma-like vascular network and positive staining for CD34 [42].

The aetiology of pleural SFT remains unknown and no association exists with smoking or asbestos exposure. The differential diagnosis for pleural SFT on histology includes other mesenchymal neoplasms characterized by a hemangiopericytoma-like vascular architecture. Previous synonyms of SFTs include fibrous mesothelioma, pleural fibroma, and sub-mesothelial fibroma. The WHO recommends that these should be discarded to avoid confusion and to denote the correct histogenesis [42].

At least 80% of all pleural SFTs are benign, while the rest may show local recurrence and metastases. Putative histological criteria for aggressive or malignant behaviour includes high mitotic activity [>4/10 HPF], high cellularity, nuclear pleomorphism and necrosis [13,42]. However, the unfavourable histological features and tumour size in themselves are unreliable harbingers of poor clinical outcome [13,17,49]. Complete initial excision remains the most important indicator of clinical outcome for both benign and malignant pleural SFTs.

### Hypoglycemia in pleural SFT

Hypoglycemia is rare in pleural SFT, occurring in approximately 5% of cases [50,51]. Doege was the first to associate this phenomenon with a pathological entity - a spindle cell neoplasm that he noted to be slow growing which he called 'fibrosarcoma of the mediastiunum' - a year before SFT was described by Klemperer and Rabin [38]. The pictures and photomicrographs in Doege's paper published in 1930 clearly show a striking resemblance to SFT [1].

Paraneoplastic hypoglycemia results from secretion of an unprocessed or incomplete high molecular weight [HMW] form of insulin-like growth factor type II [IGF-II] [52]. This HMW IGF-II is capable of activating insulin receptor thereby inhibiting hepatic gluconeogenesis and increasing peripheral glucose uptake which results in hypoglycaemia [53]. The HMW IGF-II is also capable of binding to IGF-I receptors leading to suppression of growth hormone by the pituitary, as well as reduction of insulin, IGF-I and IGF binding protein-3 by the pancreas [54].

Recently it has been shown that SFT cells have a markedly greater expression of IGF-II mRNA and a lesser expression of pro-hormone convertase 4 [PC 4] mRNA in the tumour tissue compared to normal placental tissue, raising the possibility that defective PC4 gene expression in these tumours may underlie the impaired processing of IGF-II [26]. Pro-hormone convertase 4 is an endoprotease involved in processing precursor HMW IGF-II that cleaves pro-IGF-II to generate the mature IGF-II [55,56]. The unprocessed HMW IGF-II has significantly higher bioavailability compared to mature or processed IGF-II, IGF-I and insulin, thus less able to complex serum IGF binding proteins hence its increased free levels [57,58].

The detection of HMW IGF-II requires immunoblot analysis to distinguish it from normal IGF-II; unfortunately this technology was not available to us at the time and there was no pre-operative blood specimen preserved to enable IGF-II determination. Nevertheless all the features in this case fit with the diagnosis of DPS including severe intractable hypoglycemia with suppressed insulin secretion, decreased C-peptide and immediate resolution of the hypoglycemia following excision of the tumour.

Our review confirms the notion that hypoglycemia is a rare extrathoracic manifestation of pleural SFT with less than 65 cases representing approximately 5-7% out of over 900 estimated total number of cases thus far reported in the English literature. The reported incidence is extremely variable. England *et al *reported hypoglycemia in 12 out of 223 cases [5.2%] from a multi-institutional case series [13]^3^. Rena *et al *reported hypoglycemia in 3 out 21 cases [14.3%] [5], Magdeleinat *et al *found only one case of hypoglycemia from a series of 60 cases [1.7%] [6], and Chang *et al *found one out of 14 cases [7.1%] in their series which at 33 cm also happened to be the largest tumour in that series [17]. On the other hand, Shung *et al *in a series of 63 cases [59], De Perrot *et al *in a series of 15 cases [60], and Perna *et al *with 8 cases [61], did not report a single case of hypoglycemia. As already mentioned, Briselli *et al *also did not have any single case of hypoglycemia in a series of 8 cases but established that hypoglycemia was documented in 4% of the 360 cases dating before 1981 [4].

Interestingly, onset of hypoglycemia in pleural SFT in one case coincided with the tumour growing to 13 cm over a period of 14 years of CT follow-up having grown y 5.5 cm in 7 years then 3.5 cm over another 7 years. The shortening of the tumour doubling time in these contrasting periods was suspected to be from the overproduction of tumour growth factors, including IGFII [25]. Over-expression of IGF-II mRNA is rarely observed for tumours with diameters less than 5 cm [62]. More cases of hypoglycemia occur with malignant SFT for tumours with diameters of 10 cm or more [13]. One of these cases recorded by England *et al *had fatal hypoglycemia as a direct metabolic consequence of pleural SFT [13].

The differential diagnosis of pleural based tumours presenting with hypoglycemia include other primary thoracic as well as metastatic non-islet cell epithelial and mesenchymal tumours [63]. The mesenchymal tumours include leiomyosarcoma, mesothelioma, haemangiopericytoma and the so-called malignant fibrous hustiocytoma [30,64-66]. The primary epithelial tumours include squamous cell, large cell and bronchopulmonary adenocarcinoma, while hepatocellular, gastric and exocrine pancreatic carcinomas are the common metastatic tumours to the lung that may manifest with hypoglycemia [67,68]. Some of these paraneoplastic hypoglycemias in carcinomas are attributed to IGF-I rather than IGF-II [69].

### Hypertrophic osteoarthropathy in pleural SFT

Hypertrophic osteoarthropathy (finger-clubbing) has been reported in 4-35% of patients with pleural SFT [5,6,10]. It is characterized by finger clubbing that is associated with hypertrophic skin changes, and periosteal bone changes. The paraneoplastic manifestation of finger-clubbing secondary to thoracic SFT is eponymously referred to as Pierre-Marie-Bamberg syndrome [PMBS]. Reports in literature indicate that finger-clubbing in pleural SFT is more common than hypoglycaemia [13,39,30]. The pathophysiology of finger-clubbing remains unascertained but it is postulated that the underlying HOA results from abnormal production of hyaluronic acid by the tumour cells resulting in periosteal changes, chronic hypoxia and paraneoplastic secretion of cytokines such as VEGF and PDGF. Though our patient had a history of smoking, it is the excision of the tumour that led to rapid and complete resolution of the finger-clubbing. This observation is consistent with previously published observations.

### Treatment and prognosis of pleural SFT

It is obvious from the good results reported in the literature that surgery is the treatment of choice for both malignant and benign pleural SFTs including those presenting with hypoglycemia ± finger-clubbing. Hypoglycemia and finger-clubbing almost always completely resolve following surgical excision, usually with no complications, but the symptoms may recur with recurrence of the lesion [5]. The local recurrence rate of pleural SFT following surgery is excellent for benign pleural SFT [recurrence rate 2% up to 8%] but varies widely for malignant pleural SFTs with a range of 14% for grossly pedunculated histologically malignant tumours, and up to 68% for sessile histologically malignant tumours, reflecting the importance of complete excision [39,70-72]. It is important to keep in mind that pleural SFT presenting with hypoglycemia tend to be remarkably large-sized as shown from data presented in this review (table 3). These tumours manifesting as DPS should therefore be excised intact *en bloc *with clear margins to avoid recurrence [27].

Radiotherapy and chemotherapy have limited value in the curative treatment of pleural SFT because complete surgical excision is the best treatment. Nevertheless, radiotherapy and chemotherapy have been advocated for adjuvant treatment when resection is incomplete or impossible - especially for histologically malignant tumours, as well as for recurrences, [39,73]. Neoadjuvant chemo-radiation with selective embolisation has been used successfully to reduce tumour bulk and alleviate hypoglycemia in a patient with previously irresectible abdominal SFT that responded and became amenable complete surgical resection [74]. Symptomatic medical treatment of hypoglycemia has been tried with some success for patients unfit for surgery [52,74].

Long term [5-10 years] survival rates for pleural SFT vary from 75% to 100% in various series [6,13,39,72]. Most recurrences tend to occur within 24 months of initial resection but may happen even after more than 15-20 years. All cases of pleural SFT should be followed-up with periodic chest CT scans [6 monthly to yearly] in order to monitor for recurrence, particularly those that fall in the malignant category and those that were difficult to excise [11]. Malignant transformation in recurrence of previously benign pleural SFT has been reported [75,76]. Surgical re-excision of the recurrences where possible remains the preferred treatment [77,78].

## Conclusion

Pleural SFT is an uncommon but not rare tumour that is now known to be of mesenchymal fibroblastic rather than mesothelial origin. Paraneoplastic manifestation of hypoglycemia and finger-clubbing in pleural SFT are exceedingly rare yet important clinical features in terms of diagnosis and follow-up. Hypoglycemia with or without finger-clubbing has been recorded in <80 cases in the English Literature. Both hypoglycemia and finger-clubbing resolve following excision of the tumour. In this updated review, hypoglycemia with and without finger-clubbing was found to associated with large tumours [>10 cm], but does not appear to be significantly associated with histological features of malignancy. The hypoglycemia is caused by HMW IGF-II which results from defective enzymes due to PC4 gene abnormalities in the tumour cells. The finger-clubbing results from abnormal hyaluronic acid deposition due to VEGF and PDGF cytokine expression. Histological features for malignancy and size, though important, are not truly predictive of local recurrence or worse clinical outcome. Complete surgical excision of the tumour remains the best treatment and most important predictor of clinical outcome. Long-term follow-up is recommended due to varyingly high recurrence rates particularly for tumours that were difficult to completely excise and have histological features for aggressive or malignant behaviour.

## Abbreviations

CT: Computed tomography; DPS: Doege-Potter syndrome; IGF: Insulin-like growth factor; HMW: High molecular weight; HOA: Hypertrophic osteoarthropathy; MSA: Muscle-specific acting, PDGF: Platelet-derived growth factor; PMBS: Pierre Marie-Bamberg syndrome; SFT: Solitary fibrous tumour; SMA: Smooth muscle acting; VEGF: Vascular endothelial growth factor; WHO: World Health Organisation.

## Competing interests

The authors declare that they have no competing interests.

## Authors' contributions

All authors helped to draft the manuscript, read and approved the final manuscript. AK, MJH and JM were involved in making the pathological diagnosis. MW and TH were involved in the case report, clinical workup and management of the patient. AK and JM did the literature search and review. AK coordinated the drafting of the manuscript and its preparation for publication.

## Consent Statement

Written informed consent was obtained through the University of the Witwatersrand Ethics Committee for publication of this case report and accompanying images. A copy of the written consent is available for review by the Editor-in-Chief of this journal.
